# Saponins Do Not Impede the Bioaccessibility of Minerals and Fat-Soluble Vitamins from Different Quinoa Genotypes

**DOI:** 10.1021/acs.jafc.6c01729

**Published:** 2026-04-24

**Authors:** Luise A. Lindenmayer, Miriam J. Mayer, Sandra M. Schmöckel, Jan Frank

**Affiliations:** † Institute of Nutritional Sciences, Department of Food Biofunctionality (140b), 26558 University of Hohenheim, Garbenstraße 28, 70599 Stuttgart, Germany; ‡ Institute of Crop Science, Department of Physiology of Yield Stability (340k), University of Hohenheim, Otto-Sander-Straße 5, 70599 Stuttgart, Germany; § University of Hohenheim, Cluster of Excellence, GreenRobust, 70599 Stuttgart, Germany

**Keywords:** bitter, carotenoids, Chenopodium quinoa willd., in vitro digestion, micelles, minerals, nonbitter, saponins, tocochromanols

## Abstract

Quinoa seeds contain high amounts of minerals, carotenoids, and tocochromanols (vitamin E) and can be classified into bitter and nonbitter genotypes based on their saponin contents. Saponins may form complexes with minerals and cholesterol, but their impact on the bioaccessibility of minerals, carotenoids, and tocochromanols from quinoa is unknown. We therefore subjected the seeds of 12 quinoa genotypes (*n* = 6 bitter, *n* = 6 nonbitter) to in vitro digestion and quantified the concentrations of the nutrients in digesta. Bitter and nonbitter quinoa did not differ in digestive stability, solubility, bioaccessibility, and micellar efficiency of these compounds. Mineral bioaccessibility was low, whereas that of the lipid-soluble compounds was low to medium. The mean micelle sizes and negative surface charges of mixed micelles did not differ between bitter and nonbitter quinoa seeds. In conclusion, the saponins in quinoa do not appear to affect micellar characteristics or to impair the bioaccessibility of minerals, carotenoids, and tocochromanols.

## Graphical abstract

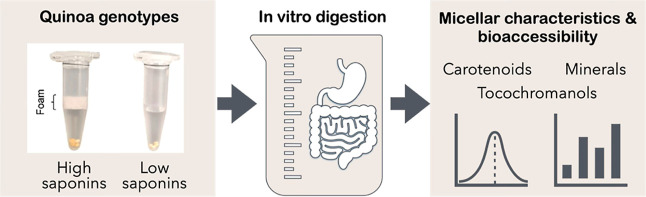

## 1 Introduction

The pseudocereal quinoa (*Chenopodium quinoa* Willd.) contains antinutrients, such as phytate and saponins.^1^ Saponins can be subdivided into triterpene and steroid glycosides and are widely distributed in the plant kingdom.^2^ Quinoa saponins are triterpene glycosides consisting of an apolar aglycone, mainly oleanolic acid, hederagenin, phytolaccagenic acid, and serjanic acid, to which polar carbohydrate chains (mostly composed of arabinose, galactose, glucose, glucuronic acid, rhamnose, and xylose) are attached.^3–6^ The saponin contents of quinoa seeds vary from 0.0 to 7.5% (by weight).^7–9^ Quinoa genotypes are thus classified into “bitter” with >0.11% and “nonbitter” with <0.11% of saponins.^9,10^

Quinoa is rich in protein, contains all essential amino acids, the minerals calcium, magnesium, iron, and zinc, and water- and lipid-soluble vitamins, including vitamin C, provitamin A-carotenoids, and tocochromanols (tocopherols and tocotrienols).^9,10^ The nutrient composition of a food alone, however, is not sufficient to characterize it as a good source of a nutrient, because dietary compounds need to be liberated from the food matrix, stable throughout digestion and soluble in chyme in order to be absorbed from the intestine.^11^ ^,^ ^12^ During digestion, lipophilic compounds, such as carotenoids and tocochromanols, are solubilized by incorporation into mixed micelles.^13^ Micellar characteristics, including size and surface charge, affect the absorption of the loaded compounds into enterocytes.^14^ In vitro digestion models have emerged as standardized and reproducible techniques for estimating the bioaccessibility of nutrients.^15^

Depending on their structure, some saponins were found to form complexes with iron and zinc, thus reducing their absorption. But so far, there is no evidence for the formation of complexes with vitamins A, E, and D_3_.^1,3,16^

Therefore, the aim of the present study was to investigate whether different saponin concentrations in seeds of bitter and nonbitter quinoa genotypes influence the stability, solubility, and bioaccessibility of minerals, carotenoids, and tocochromanols during digestion.

## 2 Materials and Methods

### 2.1 Plant Material

Seeds of 12 precharacterized quinoa genotypes (*n* = 6 bitter and *n* = 6 nonbitter; Table S1) were used for the in vitro digestion. The seeds, originating from the United States Department of Agriculture (USDA) and the Institute of Plant Genetics and Crop Science (IPK, Gatersleben, Germany), were cultivated at the Department of Physiology of Yield Stability, University of Hohenheim. These genotypes were integral to complementary research and therefore data on phenotypes and saponin contents previously published.^17,18^ Sample preparation involved milling the seeds with a high-energy vibration mill (MM 500; Retsch, Haan, Germany) at 30 Hz for 30 s, resulting in a fine, homogeneous powder. While particle size distribution was not explicitly measured, the milling parameters were chosen to ensure complete disruption of the seed coat facilitating enzymatic access during in vitro digestion.

### 2.2 Chemicals and Reagents

Butylated hydroxytoluol, 1,4-dioxane, ethanol, glacial acetic acid, *n*-hexane, potassium hydroxide (KOH, purity ≥85%), and sodium chloride (purity ≥99%) were purchased from Carl Roth GmbH & Co., KG (Karlsruhe, Germany). Methanol (MeOH) and acetonitrile (ACN) were obtained from J.T. Baker Inc. (Gliwice, Poland). Standards of carotenoids (lutein, zeaxanthin, β-carotene, β-cryptoxanthin) and tocochromanols (α-, β-, γ-, δ-tocopherol and -tocotrienol) were from Sigma-Aldrich Co. (Schnelldorf, Germany). All solvents and standards were of HPLC grade or higher quality.

### 2.3 In Vitro Digestion

Digestion experiments were performed in nine independent experiments for ten genotypes and five replicates for the remaining two due to limited sample availability (Table 1). All experiments were conducted with the same batch of enzymes with an amylase activity of 4.4 U/mg (Sigma-Aldrich, Lot BCBZ9021), pepsin activity of 484 U/mg (Sigma-Aldrich, Lot BCCJ5199), trypsin activity in pancreatin of 11.7 U/mg (Sigma-Aldrich, Lot SLCQ4385), and a bovine bile concentration of 2.83 mmol of bile salts/g (Sigma-Aldrich, Lot SLCJ0047). The enzyme activities were measured in our lab according to the INFOGEST 2.0 protocol.^15^ A blank digestion without quinoa, but with all digestion enzymes and solutions, was performed as a negative control. The in vitro digestion was performed according to the INFOGEST 2.0 protocol^15^ with slight modifications for sample amount and pancreatin solubilization according to Sousa et al.^19^ Either 200 mg (three genotypes with low amounts of sample available) or 250 mg (the remaining 9 genotypes; Table 1) of the ground quinoa seeds was diluted with 1.1 mL double-distilled water (H_2_O_dd_) to reach a theoretical food amount of 1.25 mL. Then, the quinoa paste was digested following the INFOGEST 2.0 protocol assuming it was 1.25 g of food. After adding simulated salivary fluid (1.25× conc., Table S2), CaCl_2_(H_2_O)_2_ (0.3 M), H_2_O_dd_, and amylase solution (75 U/mL in the final volume), the samples were incubated for 2 min at 37 °C in a shaking water bath (180 rpm) to simulate the oral step. For the gastric phase, simulated gastric fluid (SGF, 1.25× conc., Table S2), CaCl_2_(H_2_O)_2_ (0.3 M), and porcine pepsin solution (2000 U/mL in the final volume) were added to the oral bolus. After pH adjustment to pH 3.0 ± 0.1, H_2_O_dd_ was added to achieve a 1× concentration of SGF. The samples were incubated for 2 h at 37 °C in a shaking water bath (180 rpm). For the intestinal phase, bile (10 mM in the final volume) and pancreatin solution (trypsin activity of 100 U/mL) were both prepared in 1.25× conc. simulated intestinal fluid (SIF, Table S2) and placed in an ultrasonic bath (5 min, room temperature). Pancreatin solution was prepared as described by Sousa et al.^19^ The pancreatin solution was centrifuged (2,000*g*, 5 min, room temperature), and the supernatant transferred into a new tube and immediately used for the digestion process. After 2 h of the gastric phase, SIF (1.25× conc.), bile solution, CaCl_2_(H_2_O)_2_ (0.3 M), and freshly prepared pancreatin solution were added. Following a pH adjustment to pH 7.0 ± 0.1, H_2_O_dd_ was added to achieve a 1× concentration of SIF. After the samples were overlaid with nitrogen gas, they were incubated again for 2 h at 37 °C in a shaking water bath (180 rpm). To analyze the minerals and the fat-soluble vitamins, aliquots of 1.0 and 1.7 mL digesta of each sample were collected, respectively. The residual solutions were centrifuged (5,000*g*, 1 h, 4 °C). Volumes of 1.0 mL for minerals, 1.7 mL for lipid-soluble compounds, and 500 μL for micelle characterization were collected from the aqueous phase. The remaining portion of the aqueous phase, after collection of aliquots for mineral, lipid-soluble compounds, and micelle characterization analyses, was filtered through a 0.2 μm filter, forming the filtered aqueous phase, representing the mixed micellar fraction. As before, aliquots of 1.0 and 1.7 mL were taken for mineral and fat-soluble vitamin determination, respectively. All samples were immediately frozen after collection at −80 °C to stop enzyme activity until further analysis. Concentrations of minerals, carotenoids, and tocochromanols in quinoa seeds (raw material) were previously analyzed in our laboratory^18^ (Table S1) and the following equations were used:

$$Stability\;\left[\%\right]=\frac{concentration\;in\;digesta\;\left[mol\right]}{concentration\;in\;raw\;material\;\left[mol\right]}\times 100$$

$$Solubility\;\left[\%\right]=\frac{concentration\;in\;aqueous\;phase\;\left[mol\right]}{concentration\;in\;raw\;material\;\left[mol\right]}\times 100$$

$$Bioaccessibility\;\left[\%\right]=\frac{concentration\;in\;micellar\;fraction\;\left[mol\right]}{concentration\;in\;raw\;material\;\left[mol\right]}\times 100$$

$$Micellar\;efficiency\;\left[\%\right]=\frac{concentration\;in\;micellar\;fraction\;\left[mol\right]}{concentration\;in\;aqueous\;phase\;\left[mol\right]}\times 100$$

**Table 1:** 12 In Vitro Digested Quinoa Genotypes and Their ID, Phenotype, Number of Replication, and Amount of Quinoa Used per Digestion

genotype	ID	phenotype	number of replicates	amount of ground quinoa [mg]
CHEN-160	H00202	bitter	9	250
CHEN-389	H00203	bitter	9	250
PI-614885	H00205	bitter	9	200
PI-614888	H00311	bitter	9	250
PI-634924	67	bitter	9	250
PI-665276	667	bitter	5^a^	200
Ames-13751	147	nonbitter	5^a^	200
CHEN-126	H00204	nonbitter	9	250
CHEN-159	H00211	nonbitter	9	250
CHEN-465	H00199	nonbitter	9	250
D-12184	H00209	nonbitter	9	250
PI-510549	H00213	nonbitter	9	250

a Due to low sample availability of these genotypes, only five replicates could be made.

For minerals, only stability and solubility were calculated because solubility reflects bioaccessibility of water-soluble compounds. For lipid-soluble compounds, the bioaccessible fraction is the amount of compound incorporated into micelles, and therefore, all four parameters were calculated.

### 2.4 Analysis of Minerals in Digested Quinoa Seeds

The minerals phosphorus (P), magnesium (Mg), calcium (Ca), iron (Fe), zinc (Zn), manganese (Mn), and copper (Cu) were quantified in the seeds of the 12 quinoa genotypes after in vitro digestion via inductively coupled plasma optical emission spectrometry (ICP-OES) at the Core Facility of the University of Hohenheim according to Lauer et al.^18^

### 2.5 Analysis of Carotenoids in Digested Quinoa Seeds

The extraction of carotenoids was conducted according to Lux et al.^20^ with slight modifications according to Lauer et al.^18^ The aliquoted samples from in vitro digestion were thawed in the dark at room temperature and 800–830 μL of each sample and then saponified with 1 mL of KOH solution (50%, w/v), 1 mL of ethanol containing 0.012% β-*apo*-8′-carotenal methyloxime as an internal standard, and 1 mL of ethanol containing 0.02% butylated hydroxytoluol for 30 min in the dark in a shaking water bath at 70 °C. Then, samples were cooled on ice and 1 mL of glacial acetic acid and 2 mL of saline solution (15%, w/v) were added before extraction with *n*-hexane (2 × 2 mL, with centrifugation at 188*g*, 3 min, 4 °C). The organic phase was dried in a rotary evaporator (RVC 2–33 CDplus, Martin Christ, Osterode am Harz, Germany). The dried extract was either frozen at −80 °C until further analysis or directly dissolved in 150 μL of a 1:1 mixture of MeOH:ACN (3:7, v/v) and MeOH:ACN:1,4-dioxane (3:37:60, v/v/v). For chromatographic analysis, a Shimadzu (Kyoto, Japan) Prominence HPLC with a Reprosil 80 ODS-2 column (250 mm × 4.6 mm, 3 μm particle size, Phenomenex, Torrance, USA) at 40 °C was used. For separation, the mobile phase consisted of ACN/1,4-dioxane:MeOH (82:15:3), isocratic, at a flow rate of 1.5 mL/min in a 20 min run. The carotenoids were detected at 450 nm and quantified using a linear calibration curve containing all of the detected carotenoids and the internal standard.

### 2.6 Analysis of Tocochromanols in Digested Quinoa Seeds

The extraction of tocochromanols was conducted according to Lux et al.^20^ with slight modifications according to Lauer et al.^18^ The aliquoted samples from in vitro digestion experiments were thawed in the dark at room temperature and 800–830 μL of each sample was saponified with 2 mL ethanol containing 1% ascorbic acid, 900 μL H_2_O_dd_, and 600 μL of KOH solution (50%, w/v) for 30 min in the dark in a shaking water bath at 70 °C. Then, samples were cooled on ice, neutralized with 600 μL of glacial acetic acid, and 1 mL of H_2_O_dd_ and 25 μL of ethanol containing 1 mg/mL butylated hydroxytoluol were added. Three extractions with 2 mL of *n*-hexane following centrifugation (188*g*, 3 min, 4 °C) were performed, and the organic phase was dried in a rotary evaporator. The resulting extract was either frozen at −80 °C until further analysis or directly dissolved in 150 μL of ethanol. For chromatographic analysis, a Jasco HPLC-FL (Jasco Germany GmbH, Pfungstadt, Germany) with excitation/emission wavelengths set to 296/325 nm was used. The samples were separated within 20 min with a Kinetex F5 (150 mm × 4.6 mm, 2.6 μm particle size) column (Phenomenex, Torrance, USA) at 40 °C and MeOH:H_2_O_dd_ (87:13, v/v), isocratic, for the mobile phase at a flow rate of 1.6 mL/min. Tocochromanols were quantified using a linear calibration curve containing all eight congeners.

### 2.7 Micellar Characteristics

A Zetasizer Nano ZSP (Malvern Panalytical, Malvern, UK) was used at 25 °C, 633 nm laser wavelength, 90° scattering angle, and 173° for backscattering to quantify the size (ζ average) and surface charge (ζ potential) of mixed micelles. Three aliquots of 500 μL of the aqueous phase from each in vitro digestion experiment were filtered (0.2 μm filter) directly before measurement to obtain the mixed micellar fraction. The filtered samples were diluted 1:10 with SIF and size and surface charge of each replicate were measured immediately in a folded capillary zeta cell (Malvern Panalytical) in four consecutive runs. From dynamic light scattering, the polydispersity index (PDI) and size distribution based on the intensity of the scattered light were obtained as primary particle size data. The mean particle diameter (ζ average), and volume-weighted size distributions were derived from the intensity data.^21^

### 2.8 Statistical Analysis

All experiments were carried out using 5 or 9 repetitions for analyses of minerals and fat-soluble compounds in the in vitro digested quinoa seeds and 3 repetitions per quinoa genotype for micelle characteristics. The results are expressed as mean ± standard deviation (SD) and all data were analyzed using GraphPad Prism 10.2.0 (GraphPad software Inc., La Jolla, USA). Normal distribution of the data was computed using a Kolmogorov–Smirnov test at a significance of *p* >0.05. Differences between means of the micronutrients were calculated with one-way analysis of variance (ANOVA) using Tukey’s multiple comparison test. Differences in the micellar characteristics were calculated using an unpaired *t*-test. Significant differences between means were accepted at a *p* <0.05.

## 3 Results and Discussion

This research aimed to examine if the stability, solubility, and bioaccessibility of minerals and fat-soluble compounds are affected by the saponin content of quinoa seeds. Therefore, seeds of six saponin-rich “bitter” and six saponin-low “nonbitter” quinoa genotypes were subjected to in vitro digestion using the standardized INFOGEST 2.0 protocol. To the best of our knowledge, this is the first study to investigate the influence of quinoa saponins on the digestive stability, solubility, and bioaccessibility of minerals, carotenoids, and tocochromanols.

### 3.1 Mineral Bioaccessibility from Bitter and Nonbitter Quinoa Seeds

Minerals are water-soluble and taken up directly from the aqueous phase into enterocytes, facilitated by transporters, ion channels, and/or endocytosis and thus do not depend on micelle formation for absorption.^22^ Therefore, the concentration of minerals in the soluble phase represents the bioaccessible fraction.

The concentrations of all minerals were slightly higher in the six bitter than in the six nonbitter quinoa seeds^18^ and this was also observed after in vitro digestion (Table 2). The concentrations of minerals in the aqueous and micellar phases were similar (Tables 2 and S3). Overall, the stability and solubility of the minerals were low and did not differ between or within bitter and nonbitter quinoa, with one exception. Digestive stability of Mn in nonbitter digested quinoa was higher than its solubility (Table 2). Overall, saponins in quinoa seeds do not appear to significantly affect the bioaccessibility of these minerals.

**Table 2:** Mean (± SD) Content [mg/L], Stability, and Solubility [% of Initial Content] of Minerals in Bitter (*n* = 6) and Nonbitter (*n* = 6) Digested Quinoa Seeds.

		bitter		nonbitter	
mineral	digestive phase	[mg/L]	[%]	[mg/L]	[%]
P	stability	246.75 ± 24.05	4.76 ± 0.49	242.45 ± 8.83	5.02 ± 0.40
	solubility	226.93 ± 21.55	4.38 ± 0.46	230.59 ± 7.30	4.77 ± 0.37
Mg	stability	57.67 ± 10.10	2.18 ± 0.29	50.67 ± 5.78	2.23 ± 0.42
	solubility	49.12 ± 9.24	1.85 ± 0.27	44.81 ± 4.96	1.97 ± 0.38
Ca	stability	14.54 ± 1.77	1.32 ± 0.78	10.74 ± 3.19	0.91 ± 0.40
	solubility	6.95 ± 1.95	0.56 ± 0.23	4.64 ± 1.33	0.39 ± 0.16
Fe	stability	1.00 ± 0.24	1.23 ± 0.62	0.75 ± 0.18	1.26 ± 0.33
	solubility	0.85 ± 0.22	1.03 ± 0.52	0.66 ± 0.14	1.08 ± 0.20
Zn	stability	1.00 ± 0.20	2.82 ± 0.74	1.02 ± 0.28	2.89 ± 0.67
	solubility	0.73 ± 0.24	2.03 ± 0.67	0.69 ± 0.13	1.98 ± 0.38
Mn	stability	0.22 ± 0.16	0.52 ± 0.14	0.14 ± 0.07^a^	0.55 ± 0.25^a^
	solubility	0.13 ± 0.13	0.28 ± 0.11	0.06 ± 0.03^a^	0.22 ± 0.07^a^
Cu	stability	0.21 ± 0.03	3.07 ± 0.39	0.20 ± 0.04	2.98 ± 0.62
	solubility	0.21 ± 0.02	3.04 ± 0.38	0.21 ± 0.04	3.10 ± 0.59

a Mean contents of the minerals do not significantly differ between bitter and nonbitter genotypes. Mean values differ significantly (*p* <0.05) between stability and solubility phases of nonbitter digested seeds.

The low stability and solubility values are in accordance with the literature. In in vitro-digested raw quinoa, calcium had a low solubility of 7.35 ± 1.2%, iron of 9.10 ± 1.9%, and zinc of 14.10 ± 0.9%.^23^ These values are slightly higher than those in our study (Table 2), but they suggest that minerals overall are poorly bioaccessible from quinoa. The previous study^23^ used a different, less complex in vitro digestion protocol compared to our experiments.^15^ The differences in bioaccessibility between the two studies may also be explained by differences in the contents of saponins, (poly)­phenols, phytate, oxalates, fibers, and proteins, which can form insoluble complexes with minerals and thus inhibit their bioaccessibility.^24–28^

### 3.2 Carotenoid Bioaccessibility from Bitter and Nonbitter Quinoa Seeds

Stability, solubility, and bioaccessibility of carotenoids from in vitro digested quinoa seeds were low to medium. While lutein, zeaxanthin, and β-carotene were quantified in all 12 genotypes, β-cryptoxanthin was only detectable in four (two bitter and two nonbitter) of the stability samples and in two (one bitter and one nonbitter) of the soluble fractions. In all micellar fractions, β-cryptoxanthin was below the limit of detection. This can most likely be explained by the initially low contents of β-cryptoxanthin in ripe quinoa seeds.^18^ Hence, only mean stability and solubility could be calculated and bioaccessibility of β-cryptoxanthin was assumed to be 0.00%. Therefore, all values for β-cryptoxanthin were significantly lower than for the other carotenoids with mean values ranging between 0.00% and 13.56 ± 3.00% (PI-510549; Table S4). No differences in the digestion parameters (stability, solubility, bioaccessibility, and micellar efficiency) between bitter and nonbitter quinoa genotypes were observed for any of the carotenoids (Figure 1) except for the micellar efficiency of zeaxanthin and β-carotene, which were higher in bitter than nonbitter quinoa seeds (Figure 1b,1c and Table S4). Despite some variability within the data set, no consistent trend between genotype groups was observed. Therefore, quinoa saponins do not seem to affect the bioaccessibility of these carotenoids.

**Figure 1:**
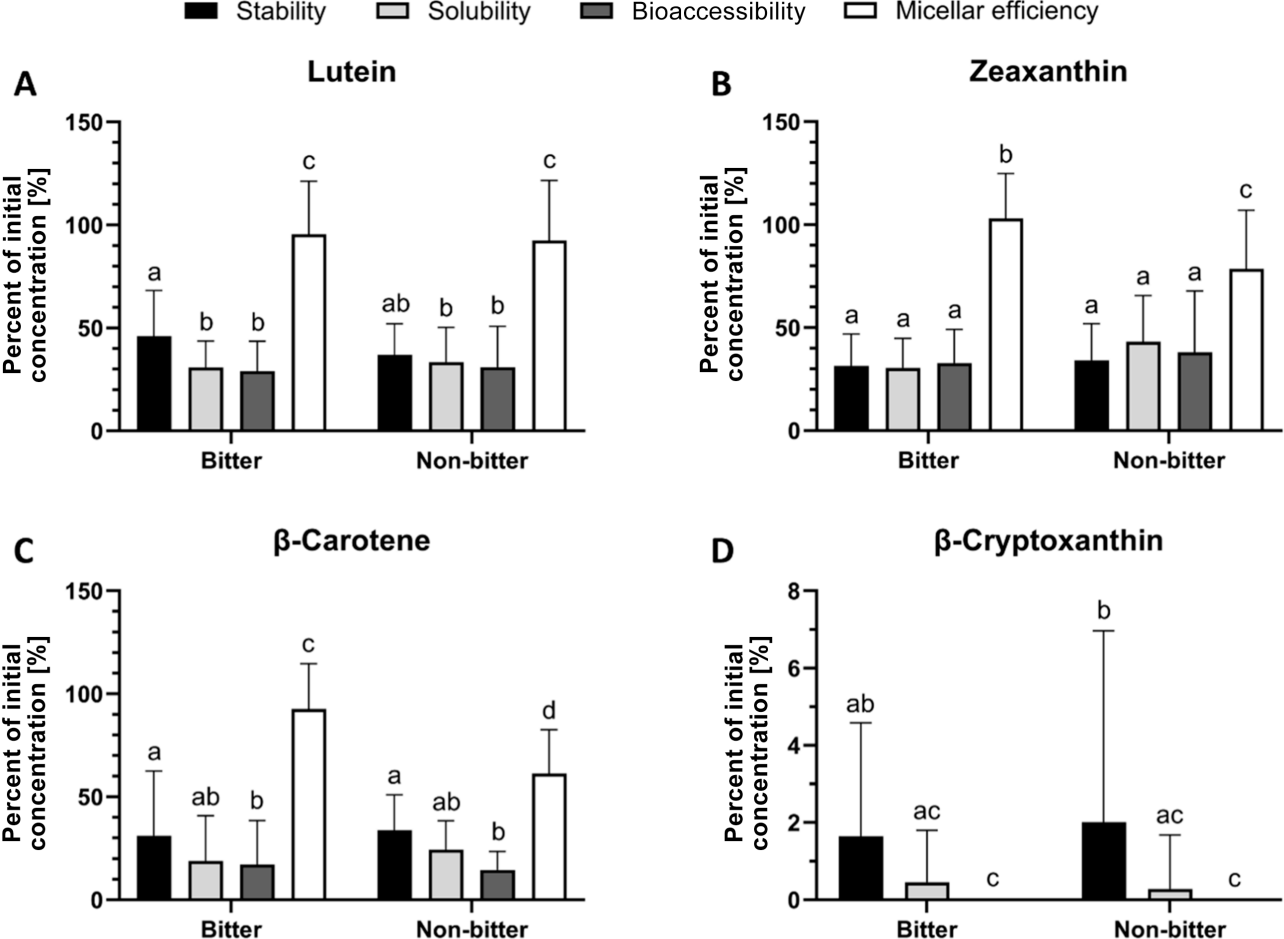
Differences in carotenoid stability, solubility, bioaccessibility, and micellar efficiency [%] between bitter and nonbitter quinoa genotypes after in vitro digestion. Results are expressed as mean ± SD (*n* = 6 bitter, *n* = 6 nonbitter quinoa genotypes). Values not sharing a common superscript letter significantly differ at *p* <0.05; one-way ANOVA with Tukey’s posthoc test.

These observations are supported by the fact that neither of the single saponins (digitonin, ammoniated glycyrrhizin) nor of saponin mixtures (quillaja bark, alfalfa root and leaf, soybean saponins) form complexes with vitamin A.^29^ Based on the structural similarities of vitamin A and carotenoids, this may also apply to carotenoids. However, a recent in vitro digestion study with *Phaseolus vulgaris L.* beans revealed a slight inverse correlation between saponin contents and solubility of lycopene, phytoene, and β-carotene.^30^

After in vitro digestion of pseudocereal-enriched water biscuits consisting of 70% einkorn and 30% quinoa, solubilities of 82% for lutein, 79% for zeaxanthin, 100% for α- and β-carotene combined, and 71% for β-cryptoxanthin were reported,^31^ which exceed those observed in our present study (lutein, 32%; zeaxanthin, 37%; β-carotene, 22%; and β-cryptoxanthin, 0.4%, Table S4).

The bioaccessibility of lutein and zeaxanthin from the 12 quinoa genotypes mostly correspond to values observed in different bean landraces and corn cereals.^32,33^ The low stability at gastric acidic pH, a negative correlation of lutein with phytic acid and zeaxanthin with dietary fiber,^32^ and the inverse correlation of both carotenoids with fiber and anthocyanins were proposed to reduce carotenoid bioaccessibility.^33^ Thus, differences in the contents of these compounds, rather than in saponin contents, might explain the differences in bioaccessibilities observed in the genotypes analyzed.

### 3.3 Tocochromanol Bioaccessibility from Bitter and Nonbitter Quinoa Seeds

The tocochromanols showed mostly medium-to-high values for stability and micellar efficiency and low-to-medium solubilities and bioaccessibilities with the exception of αT, which had low digestive stability, solubility, and bioaccessibility across all genotypes (Figure 2 and Table S5). The other six tocochromanols were over 2- to 7-times more bioaccessible compared to αT (Figure 2 and Table S5). After in vitro digestion, βT could not be detected in any genotype (Table S5). The high standard deviations of γT3 (Figure 2f) were observed because it was detected in only one-third of the bitter genotypes (CHEN-160, CHEN-389) and in half of the nonbitter genotypes (CHEN-126, CHEN-465, PI-510549; Table S5), but the mean was calculated for all 6 bitter and nonbitter genotypes, respectively. Calculating the mean only for the samples where γT3 was detected, stability and micellar efficiency ranged from 89 to 103%, solubility and bioaccessibility from 49 to 58%. Despite the observed variability in some parameters, there were no consistent or systematic differences in the digestion parameters (stability, solubility, bioaccessibility, micellar efficiency) between bitter and nonbitter genotypes except for the stability of γT, δT, βT3, and δT3 (bitter > nonbitter) and the solubility of αT3 (bitter < nonbitter) (Figure 2). Although the stabilities of 4 out of 7 detected tocochromanols were higher in bitter quinoa seeds, the tocochromanol concentrations in the more relevant micellar fractions did not differ between bitter and nonbitter quinoa genotypes (Figure 2). Thus, saponins may play only a minor role in the digestion of tocochromanols.

**Figure 2:**
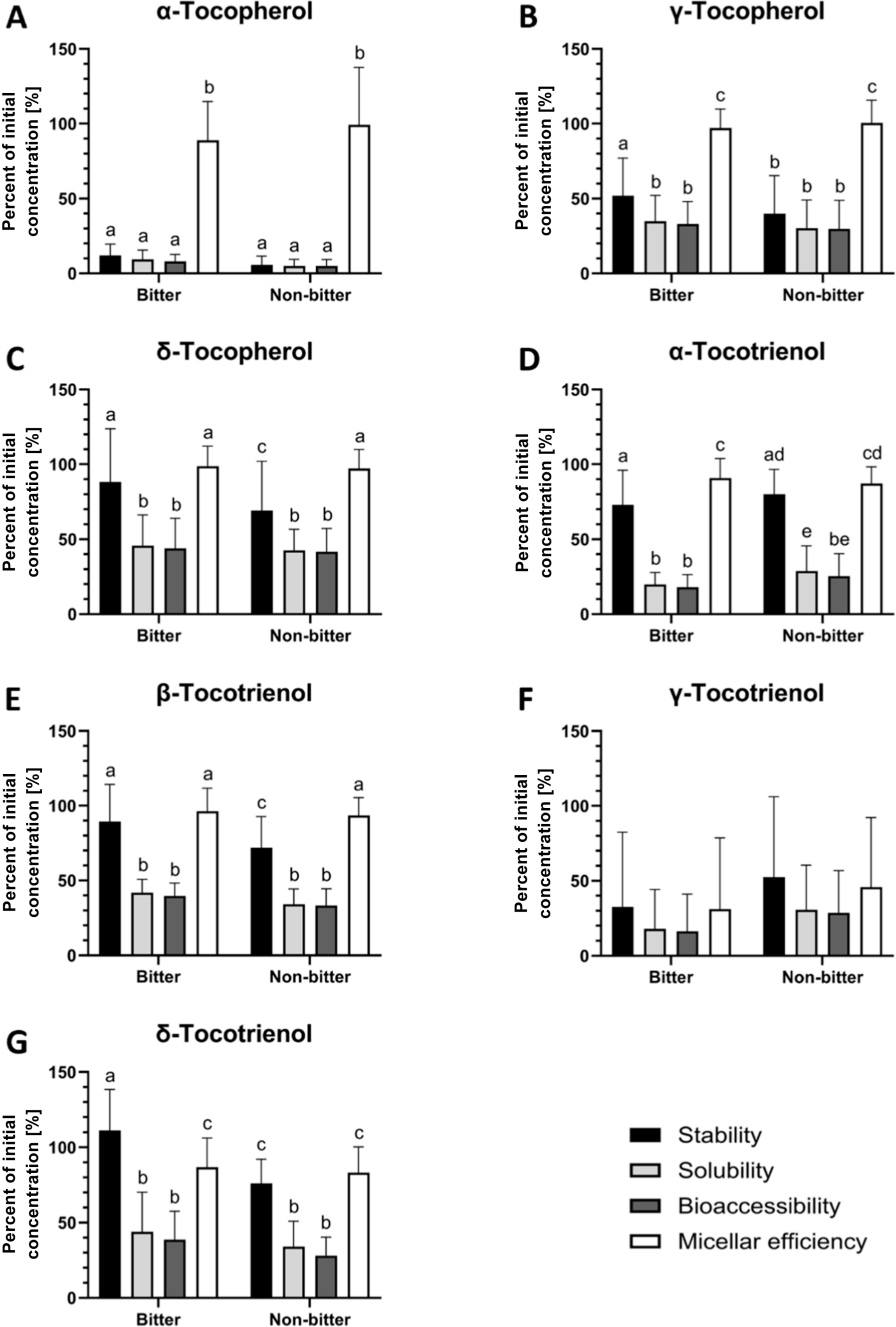
Differences in tocochromanol stability, solubility, bioaccessibility, and micellar efficiency [%] between bitter and nonbitter quinoa genotypes after in vitro digestion. Results are expressed as mean ± SD (*n* = 6 bitter, *n* = 6 nonbitter quinoa genotypes). Values not sharing a common superscript letter significantly differ at *p* <0.05; one-way ANOVA with Tukey’s posthoc test.

The saponins digitonin, ammoniated glycyrrhizin, and saponin mixtures from quillaja bark, alfalfa root, and leaf and soybean do not form complexes with vitamin E,^29^ which is in accordance with the results of the present study.

After in vitro digestion of pseudocereal-enriched water biscuits, the solubilities of tocochromanols except δT, γT3, and δT3 were analyzed previously.^31^ All solubility values were substantially higher (e.g., αT showed a 10-fold higher solubility of 75.33%) than those observed in the present study (Figure 2 and Table S5). While βT was not detected in any quinoa genotype in the current study, it was highly soluble in the biscuits (77.27%).^31^ Similar to these findings, βT from the hydrolyzed quinoa extract showed a bioaccessibility of around 70%.^12^ Conversely, δT was moderately soluble in our study (43.56% for all 12 genotypes) yet undetectable in the biscuits.^31^ Further emphasizing this variability, the bioaccessibility of quantified tocopherols in four differently colored raw quinoa genotypes was reported to be significantly lower (around 2%) than in our experiments.^34^ These differences are likely attributable to variations in the quinoa genotypes evaluated and the specific in vitro digestion protocol. Although the water biscuits^31^ and the raw quinoa seeds^34^ utilized the same digestion protocol,^35^ the final enzyme and bile concentrations for the quinoa seeds were 12.5- to 20-fold higher than in the biscuits and in our study. The hydrolyzed quinoa extracts were digested using a more simplified digestion method.^12^ Furthermore, the product matrix differed substantially (e.g., 30% quinoa in biscuits vs 100% raw seeds or extracts) as did initial tocochromanol concentrations, introducing potential concentration-dependent effects. The bioaccessibilities of γT and δT from the 12 quinoa genotypes mostly correspond to those observed in bean landraces.^32^ As tocochromanol bioaccessibility is highly influenced by the release of constituents from the food matrix, food processing, as well as fibers, fats, and phytosterols,^32^ differences in the contents of these compounds, rather than saponin contents, might explain the variations in bioaccessibilities across the analyzed genotypes.

### 3.4 Micellar Characteristics

While the digestive end-products of hydrophilic compounds, such as carbohydrates, proteins, and minerals, are directly absorbed by specific transport proteins, lipophilic compounds, such as carotenoids and tocochromanols, depend on their incorporation into mixed micelles for solubilization in the chyme and transport through the intestinal brush border membrane.^13^ Therefore, micellar characteristics, namely, polydispersity index (PDI), particle size (ζ average), and surface charge (ζ potential) of mixed micelles, were measured after in vitro digestion of quinoa seeds. The PDI of mixed micelles was between 0.34 and 0.66 (Table S6) and did not differ between bitter and nonbitter genotypes (Table 3). The dimensionless PDI describes the heterogeneity of a particle size distribution and can reach values between 0 and 1.^36^ Values of <0.1 represent a highly monodisperse system, values between 0.1 and 0.7 a nearly monodisperse system, and >0.7 a polydisperse system with a very broad distribution of particle sizes.^21,36^ Since our mean PDI was below 0.7, the solutions were nearly monodisperse to moderately polydisperse.

**Table 3:** Mean (± SD) PDI, ζ Average [nm], and ζ Potential [mV] in Bitter (*n* = 6) and Nonbitter (*n* = 6) Digested Quinoa Seeds^a^

	bitter	nonbitter
PDI	0.57 ± 0.02	0.52 ± 0.06
ζ average [nm]	145.61 ± 16.82	204.20 ± 62.46
ζ potential [mV]	–26.35 ± 0.22	–26.91 ± 2.12

a Means do dot significantly differ between bitter and nonbitter genotypes.

Slightly but nonsignificantly higher ζ average of nonbitter compared to bitter quinoa mixed micelles was observed (Tables 3, S6). Particles with ζ potentials between −10 and +10 mV are approximately neutral. Strongly anionic or strongly cationic particles have a surface charge of less than −30 or higher than +30 mV, respectively.^37^ The particles of all 12 genotypes showed a negative surface charge, which is most likely attributable to the negatively charged bile salts incorporated into mixed micelles.^38^ The micelles generated during digestion of all genotypes had a mean surface charge between −24.00 and −28.79 mV except CHEN-465, which had a strongly anionic charge of −30.39 mV (Table S6). However, no significant differences in the ζ potential were found between the bitter and nonbitter genotypes (Table 3), suggesting that the saponins had no impact on the size and surface charges of the micelles.

The volume-weighted distribution provides the relative proportion of different particle sizes in a sample based on their volume.^21^ Although the samples were filtered through a 0.2 μm filter, a large fraction of the particles exceeded this filtration limit and exhibited particle sizes greater than 200 nm (from 15.89% in D-12184 up to 81.89% in CHEN-465; Figure 3). This seems to be more pronounced for particles formed during the digestion of the nonbitter than of the bitter genotypes, with 54.11 ± 23.08% of the nonbitter and 44.56 ± 10.00% of the bitter particles above the filter cutoff, respectively. This may be explained by the aggregation of smaller micelles into larger particles in the time between filtration and measurement of the samples.^14^ Particle sizes in the digesta of both bitter and nonbitter genotypes showed a peak at 5560 nm with 12.18% for bitter and 5.80% for nonbitter genotypes. Nonbitter samples showed an additional peak between 458.7 and 712.4 nm (3.43–3.64% with the peak at 615.1 nm). Most of the particles below the cutoff had the size of 58.77 nm (2.85% in bitter, 2.51% in nonbitter genotypes, Figure 3).

**Figure 3:**
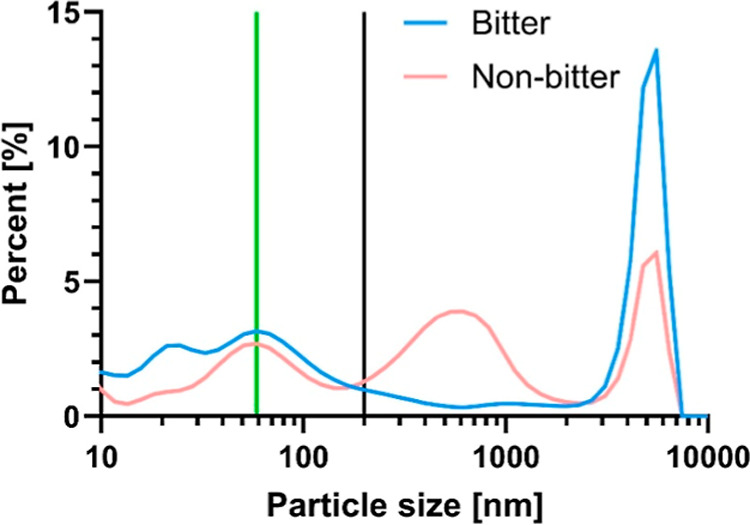
Volume distribution [%] of the particles [nm] formed during in vitro digestion of bitter and nonbitter genotypes on a logarithmic scale with the green line showing the peak of both bitter and nonbitter genotypes at 58.77 nm, and the black line reflecting the filter cutoff at 200 nm. Results are expressed as mean ± SD (*n* = 6 bitter, *n* = 6 nonbitter quinoa genotypes).

Previously, an increasing β-carotene bioaccessibility was observed with a smaller lipid droplet size because rising droplet sizes resulted in a lower rate of lipid digestion.^39^ This resulted in a bioaccessibility of 82.5% for a particle diameter of 160 nm and 46.5% for 1090 nm, respectively. Considering a decreased intestinal absorption of larger particles,^39^ this could imply that the lipid-soluble carotenoids and tocochromanols might be slightly better bioaccessible from bitter quinoa genotypes compared to nonbitter genotypes and, in turn, better absorbed. But taking all micelle parameters into account, only the volume distribution of the micelles varied a little between the bitter and nonbitter group. Therefore, these variations do not seem to be large enough to result in differences in the bioaccessibility of carotenoids and tocochromanols between bitter and nonbitter quinoa genotypes.

While the applied in vitro digestion model provides valuable insights into the bioaccessibility of nutrients, it does not fully reflect the complexity of human digestion and absorption processes. In particular, physiological factors such as intestinal transport mechanisms, metabolism within enterocytes, and interindividual variability are not accounted for. Furthermore, interactions with the gut microbiota, which may influence the transformation of saponins and other bioactive compounds, are not represented in this model.^15^

Therefore, the results obtained in this study should be interpreted as an estimation of potential bioaccessibility rather than actual bioavailability in vivo. Further studies using cell-based models or in vivo approaches are required to confirm the physiological relevance of the observed findings.

To conclude, this is the first study to investigate the impact of quinoa saponins on the bioaccessibility of minerals (P, Mg, Ca, Fe, Zn, Mn, and Cu) and lipid-soluble carotenoids and tocochromanols. Contrary to their reputation as antinutrients, our findings demonstrate no adverse effects of saponins on nutrient bioaccessibility. This observation is further supported by the micellar characteristics examined, which mechanistically confirm that the presence of quinoa saponins in saponin-rich bitter quinoa genotypes does not interfere with nutrient bioaccessibility throughout in vitro digestion compared to saponin-poor nonbitter quinoa genotypes. Hence, our findings suggest that bitterness is not an exclusion criterion for the cultivation or consumption of these genotypes. Consequently, agricultural breeding programs can continue to prioritize traits such as pest resistance and climate resilience—often associated with higher saponin levels^40^—without compromising the delivery of essential micronutrients to the consumer.

## Supplementary Material



## Notes

Additional experimental details; quinoa genotypes and composition; composition of digestion fluids (SSF, SGF, SIF); detailed digestion parameter data for minerals, carotenoids, and tocochromanols; and detailed micellar characteristics (PDF)

## Abbreviations

ACN: acetonitrile
ANOVA: analysis of variance
Ca: calcium
Cu: copper
Fe: iron
H_2_O_dd_: double-distilled water
ICP-OES: inductively coupled plasma optical emission spectrometry
KOH: potassium hydroxide
MeOH: methanol
Mg: magnesium
Mn: manganese
P: phosphorus
PDI: polydispersity index
T: tocopherol
T3: tocotrienol
Zn: zinc
