# Supplementary material for: Saponins Do Not Impede the Bioaccessibility of Minerals and Fat-Soluble Vitamins from Different Quinoa Genotypes
Source: J Agric Food Chem. 2026 Apr 24;74(17):13702–10. doi: 10.1021/acs.jafc.6c01729 (PMC13154180; doi:10.1021/acs.jafc.6c01729)
Supplement: Supplementary file 1 [file jf6c01729_si_001.pdf]

Supporting Information

Saponins do not impede the bioaccessibility of minerals and fat-soluble vitamins from different quinoa genotypes

Luise A. Lindenmayer,[a] Miriam J. Mayer,[a] Sandra M. Schmöckel,[b,c] Jan Frank [a]*

[a] University of Hohenheim, Institute of Nutritional Sciences, Department of Food Biofunctionality (140b), Garbenstraße 28, 70599 Stuttgart, Germany

[b] University of Hohenheim, Institute of Crop Science, Department of Physiology of Yield Stability (340k), Otto-Sander-Straße 5, 70599 Stuttgart, Germany

[c] University of Hohenheim, Cluster of Excellence, GreenRobust, 70599 Stuttgart, Germany

* Adress correspondence to: jan.frank@nutres.de

Table S1. Selection of twelve quinoa genotypes for *in vitro* digestion with their ID, phenotype, site, country of origin and micronutrients

Genotype	ID	Phenotype	Site	Country of origin	Saponins [mg/100 g] [a]	Minerals [mg/kg] [b]	[illegible]
CHEN-160	H00202	Bitter	highland	Bolivia	639.11	8405.64	
CHEN-389	H00203	bitter	highland	Peru	807.57	9859.45	
PI-614885	H00205	bitter	lowland	Chile	691.11	13499.19	
PI-614888	H00311	bitter	lowland	Chile	480.73	6800.71	
PI-634924	67	bitter	lowland	Chile	648.96	9465.15	
PI-665276	667	bitter	highland	Bolivia	-	10125.31	
Ames-13751	147	non-bitter	lowland	USA	<10	7364.26	
CHEN-126	H00204	non-bitter	highland	Bolivia	<10	8813.23	
CHEN-159	H00211	non-bitter	highland	Bolivia	<10	7418.80	
CHEN-465	H00199	non-bitter	highland	Peru	<10	8654.16	
D-12184	H00209	non-bitter	highland	Peru	<10	8746.48	
PI-510549	H00213	non-bitter	highland	Peru	<10	10867.88	

[a] Kollmar, M.; Böndel, K. B.; John, L.; Arold, S.; Schmid, K.; Jarvis, D.; Schmöckel, S. M.; Otterbach, S. L. Investigating the bHLH transcription factor TSARL1 as marker and regulator of saponin biosynthesis in Chenopodium quinoa. *Journal of the Science of Food and Agriculture* **2025.** DOI: 10.1002/jsfa.14436.

[b] Lauer, L. A.; Kollmar, M.; Schmöckel, S. M.; Frank, J. Comparative analysis of minerals, carotenoids, and tocochromanols in ripe seeds, immature seeds and tepals of bitter and non-bitter quinoa genotypes. *Journal of Food Composition and Analysis* **2026**, *152*, 109021. DOI: 10.1016/j.jfca.2026.109021.

Tabe S2. Composition of simulated salivary fluid (SSF), simulated gastric fluid (SGF) and simulated intestinal fluid (SIF) used for the in vitro digestion

Salt	Stock solution to prepare [g/100 mL]	SSF (pH 7) 1.25x		SGF (pH 3) 1.25x		SIF (pH 7) 1.25x	
		mL to add for a final volume of 200 mL	Final concentration in SSF [mM]	mL to add for a final volume of 400 mL	Final concentration in SGF [mM]	mL to add for a final volume of 400 mL	Final concentration in SIF [mM]
**KCl**	3.73	7.55	15.1	6.9	6.9	6.8	6.8
**KH_2PO_4**	6.8	1.85	3.7	0.9	0.9	0.8	0.8
**$NaHCO_3$**	8.4	3.4	13.6	12.5	25	42.5	85
**NaCl**	11.7	-	-	11.8	47.2	9.6	38.4
**$MgCl_2(H_2O)_6$**	3.05	0.25	0.15	0.4	0.12	1.1	0.33
**$(NH_4)_2CO_3$**	4.8	0.03	0.06	0.5	0.5	-	-
**HCl**	-	0.045	1.1	1.3	15.6	0.7	8.4

Table S3. Mean (± standard deviation) contents [mg/l] and bioaccessibility [% of initial content] of the mixed micellar fraction of minerals in bitter (n=6) and non-bitter (n=6) digested quinoa seeds. Mixed micellar fraction content and resulting percentages were equal to the values obtained in the aqueous phase (Table 2). Therefore, the aqueous phase of minerals can not only be considered as soluble phase but also as bioaccessible phase.

**Mineral**	**Bitter**		**Non-bitter**	
	**[mg/l]**	**[% of initial content]**	**[mg/l]**	**[% of initial content]**
**P**	226.40 ± 21.06	4.38 ± 0.48	231.22 ± 6.40	4.79 ± 0.44
**Mg**	48.59 ± 8.83	1.84 ± 0.26	45.21 ± 4.99	1.99 ± 0.41
**Ca**	5.77 ± 2.38	0.45 ± 0.18	2.69 ± 1.21	0.23 ± 0.13
**Fe**	0.84 ± 0.23	1.01 ± 0.51	0.64 ± 0.18	1.04 ± 0.23
**Zn**	0.73 ± 0.25	2.04 ± 0.71	0.70 ± 0.14	1.99 ± 0.35
**Mn**	0.13 ± 0.13	0.27 ± 0.12	0.06 ± 0.03	0.21 ± 0.07
**Cu**	0.21 ± 0.02	3.04 ± 0.34	0.21 ± 0.04	3.07 ± 0.60

Table S4. Stability, solubility, bioaccessibility and micellar efficiency [% of initial content] of carotenoids in digested quinoa

	**Digestion parameters**	**Bitter**							[illegible]		
		CHEN-160	CHEN-389	PI-614885	PI-614888	PI-634924	PI-665276	**Mean**	Ames-13751	CHEN-126	[illegible]
Lutein	Stability	32.78 ± 2.33	28.23 ± 1.68	82.84 ± 9.55	58.60 ± 4.85	45.74 ± 2.41	24.49 ± 4.99	45.45 ± 20.28	52.78 ± 6.25	15.17 ± 1.72	[illegible]
	Solubility	30.66 ± 6.31	17.86 ± 0.89	48.52 ± 4.45	26.50 ± 10.21	40.77 ± 2.96	13.96 ± 0.46	29.71 ± 12.08	44.35 ± 7.44	12.43 ± 1.70	[illegible]
	Bioaccessibility	29.23 ± 2.81	15.97 ± 0.68	53.47 ± 11.90	27.99 ± 5.22	25.89 ± 6.72	15.69 ± 0.82	28.04 ± 12.59	39.47 ± 0.79	12.31 ± 1.21	[illegible]
	Micellar efficiency	99.94 ± 24.92	89.73 ± 7.01	103.09 ± 13.88	104.54 ± 36.23	56.34 ± 10.01	112.48 ± 5.54	94.36 ± 18.29	88.06 ± 14.09	100.18 ± 12.28	[illegible]
Zeaxanthin	Stability	27.08 ± 7.07	14.80 ± 2.46	62.31 ± 6.48	30.49 ± 6.56	39.11 ± 5.89	3.34 ± 5.15	29.52 ± 18.60	33.10 ± 3.59	24.27 ± 3.23	[illegible]
	Solubility	35.03 ± 4.85	7.99 ± 1.27	44.06 ± 10.62	40.83 ± 8.13	29.75 ± 8.78	20.22 ± 3.84	29.56 ± 16.92	45.25 ± 5.27	26.49 ± 3.33	[illegible]
	Bioaccessibility	45.05 ± 3.19	7.57 ± 0.60	49.12 ± 7.21	43.10 ± 7.72	24.11 ± 3.21	20.07 ± 1.66	31.50 ± 15.20	37.84 ± 6.04	20.62 ± 6.48	[illegible]
	Micellar efficiency	114.78 ± 15.69	96.00 ± 14.27	108.04 ± 19.88	103.63 ± 24.46	91.96 ± 26.25	102.30 ± 17.50	102.79* ± 15.51	84.31 ± 12.93	77.28 ± 17.17	[illegible]
β-Carotene	Stability	12.57 ± 1.72	16.51 ± 3.30	16.05 ± 0.56	95.27 ± 11.25	29.15 ± 3.12	12.15 ± 1.56	30.28 ± 29.61	34.54 ± 1.01	4.59 ± 0.70	[illegible]
	Solubility	5.90 ± 0.51	8.68 ± 0.97	8.70 ± 0.97	63.76 ± 8.57	19.16 ± 2.72	5.63 ± 0.70	20.30 ± 20.68	24.91 ± 4.40	2.79 ± 0.50	[illegible]
	Bioaccessibility	4.63 ± 0.59	6.67 ± 1.11	8.46 ± 2.15	63.95 ± 7.81	13.27 ± 7.94	4.81 ± 0.41	16.97 ± 21.21	20.11 ± 1.28	1.79 ± 0.64	[illegible]
	Micellar efficiency	77.81 ± 11.64	82.14 ± 12.41	97.59 ± 22.70	113.55 ± 23.70	101.61 ± 12.87	86.50 ± 10.42	93.20* ± 12.30	73.61 ± 4.21	66.99 ± 17.55	[illegible]
β-Cryptoxanthin	Stability	5.09 ± 0.85	ND	ND	ND	ND	7.95 ± 1.89	1.64 ± 2.91	ND	ND	[illegible]
	Solubility	4.20 ± 2.14	ND	ND	ND	ND	ND	0.46 ± 1.32	ND	ND	[illegible]
	Bioaccessibility	ND	ND	ND	ND	ND	ND	0.00	ND	ND	[illegible]
	Micellar efficiency	ND	ND	ND	ND	ND	ND	ND	ND	ND	[illegible]

ND: not detected. *Mean values differ significantly ($p<0.05$) between bitter and non-bitter seeds.

Table S5. Stability, solubility, bioaccessibility and micellar efficiency [% of initial content] of tocochromanols in digested quinoa

	Digestion parameters	Bitter							Non-bitter						
		CHEN-160	CHEN-389	PI-614885	PI-614888	PI-634924	PI-665276	**Mean**	Ames-13751	CHEN-126	CHEN-159	CHEN-465	D-12184	PI-510549	**Mean**
αT	Stability	9.09 ± 3.39	5.32 ± 1.29	18.53 ± 3.10	22.81 ± 2.64	7.00 ± 2.93	5.18 ± 2.98	11.32 ± 6.85	18.47 ± 1.66	8.34 ± 2.51	0.14 ± 0.25	ND	4.45 ± 0.72	8.81 ± 2.15	6.70 ± 6.31
	Solubility	6.15 ± 1.61	6.80 ± 1.61	12.61 ± 1.80	19.74 ± 3.86	5.44 ± 1.43	1.68 ± 0.55	8.76 ± 5.88	15.50 ± 1.84	5.23 ± 2.74	1.47 ± 0.72	1.20 ± 0.46	3.73 ± 0.58	7.34 ± 1.15	5.74 ± 4.85
	Bioaccessibility	5.42 ± 1.40	4.49 ± 1.06	12.09 ± 2.42	14.99 ± 1.19	6.06 ± 1.21	2.77 ± 0.51	7.64 ± 4.38	16.16 ± 2.31	4.38 ± 2.79	1.69 ± 0.80	2.39 ± 0.64	3.63 ± 0.99	5.71 ± 0.94	5.66 ± 4.87
	Micellar efficiency	77.74 ± 20.01	71.89 ± 11.82	87.62 ± 12.29	78.37 ± 13.63	106.17 ± 19.09	149.81 ± 4.73	95.27 ± 26.74	98.54 ± 16.67	69.13 ± 18.36	98.11 ± 16.29	181.11 ± 6.07	98.83 ± 18.46	75.78 ± 13.52	103.58 ± 36.63
βT	Stability	ND	ND	ND	ND	ND	ND	ND	ND	ND	ND	ND	ND	ND	ND
	Solubility	ND	ND	ND	ND	ND	ND	ND	ND	ND	ND	ND	ND	ND	ND
	Bioaccessibility	ND	ND	ND	ND	ND	ND	ND	ND	ND	ND	ND	ND	ND	ND
	Micellar efficiency	ND	ND	ND	ND	ND	ND	ND	ND	ND	ND	ND	ND	ND	ND
γT	Stability	20.32 ± 2.40	42.57 ± 0.53	62.85 ± 6.70	91.25 ± 7.16	57.10 ± 11.54	25.91 ± 8.40	50.00* ± 23.92	95.90 ± 5.77	56.09 ± 8.05	29.64 ± 3.05	9.78 ± 1.30	27.65 ± 3.35	49.19 ± 7.41	44.71* ± 27.41
	Solubility	10.29 ± 0.83	33.28 ± 3.30	41.06 ± 2.60	60.04 ± 5.34	41.09 ± 6.30	15.47 ± 0.24	33.54 ± 16.75	74.64 ± 5.37	35.84 ± 4.43	28.02 ± 4.01	9.76 ± 1.76	18.22 ± 2.07	36.03 ± 4.42	33.75 ± 20.54
	Bioaccessibility	10.11 ± 0.63	31.03 ± 3.24	37.79 ± 2.27	51.78 ± 1.71	43.58 ± 4.18	16.60 ± 0.73	31.81 ± 14.58	76.46 ± 4.33	33.01 ± 9.79	28.37 ± 3.83	11.74 ± 1.61	17.40 ± 3.86	33.25 ± 3.56	33.37 ± 20.83
	Micellar efficiency	98.86 ± 10.07	93.81 ± 10.44	94.60 ± 7.21	86.95 ± 8.31	108.04 ± 15.86	102.84 ± 5.23	97.51 ± 6.77	98.47 ± 5.30	91.82 ± 14.81	103.41 ± 14.04	119.25 ± 10.73	94.05 ± 9.44	91.67 ± 3.74	99.78 ± 9.63

δT	Stability	71.83 ± 12.18	158.25 ± 18.01	82.54 ± 8.46	99.63 ± 7.02	71.76 ± 4.40	39.15 ± 7.97	87.19* ± 36.53	84.41 ± 5.00	58.46 ± 6.25	79.88 ± 8.38	130.84 ± 3.60	33.19 ± 3.94	47.06 ± 10.66	72.31* ± 31.59
	Solubility	25.28 ± 1.48	81.37 ± 6.45	33.94 ± 3.26	51.84 ± 2.92	48.81 ± 7.06	23.09 ± 1.52	44.05 ± 19.87	50.42 ± 3.58	40.62 ± 4.68	51.79 ± 4.71	60.54 ± 4.21	20.82 ± 1.49	34.18 ± 3.96	43.06 ± 13.01
	Bioaccessibility	24.20 ± 1.25	78.83 ± 6.40	28.37 ± 3.39	49.05 ± 2.89	53.97 ± 4.14	24.24 ± 1.54	43.11 ± 19.83	52.48 ± 2.25	35.91 ± 2.97	52.02 ± 2.84	63.16 ± 3.08	19.03 ± 3.48	33.63 ± 2.28	42.71 ± 14.66
	Micellar efficiency	96.06 ± 7.70	95.12 ± 12.57	88.00 ± 9.84	96.72 ± 3.75	112.53 ± 15.61	105.39 ± 9.17	98.66 ± 7.89	100.11 ± 3.75	89.63 ± 9.80	101.25 ± 13.26	104.73 ± 7.45	91.71 ± 17.54	96.66 ± 7.78	97.35 ± 5.31
αT3	Stability	74.34 ± 23.25	86.00 ± 5.98	103.28 ± 14.23	71.15 ± 6.12	48.31 ± 10.51	50.85 ± 5.24	72.32 ± 19.10	54.22 ± 8.63	87.37 ± 7.21	75.10 ± 14.31	72.65 ± 6.98	82.58 ± 12.18	96.89 ± 11.20	78.13 ± 13.34
	Solubility	9.37 ± 0.53	18.31 ± 0.56	15.81 ± 0.84	29.49 ± 2.13	29.50 ± 3.21	13.64 ± 1.68	19.35* ± 7.65	23.93 ± 2.33	23.15 ± 2.11	16.55 ± 2.34	62.77 ± 5.63	21.39 ± 1.63	22.51 ± 2.89	28.38* ± 15.56
	Bioaccessibility	7.16 ± 0.36	16.47 ± 1.28	12.89 ± 1.21	25.93 ± 2.38	29.99 ± 3.00	14.03 ± 0.83	17.75 ± 7.83	25.86 ± 4.02	18.97 ± 1.10	13.84 ± 0.79	54.28 ± 4.59	17.73 ± 2.17	19.80 ± 2.30	25.08 ± 13.53
	Micellar efficiency	76.74 ± 6.92	89.95 ± 6.24	84.23 ± 8.32	90.56 ± 8.50	102.91 ± 15.49	99.22 ± 7.10	90.60 ± 8.75	101.99 ± 3.18	82.94 ± 9.37	83.97 ± 11.78	86.89 ± 7.94	86.98 ± 10.74	87.59 ± 11.70	88.39 ± 6.31
βT3	Stability	119.28 ± 22.98	83.44 ± 11.12	91.38 ± 15.00	104.15 ± 8.38	63.18 ± 10.81	64.11 ± 11.19	87.59* ± 20.24	74.21 ± 5.91	119.59 ± 10.97	64.48 ± 9.05	63.68 ± 4.29	66.87 ± 5.23	58.77 ± 10.78	76.60* ± 20.64
	Solubility	47.88 ± 5.54	35.12 ± 3.92	37.68 ± 5.14	52.07 ± 4.18	42.48 ± 5.56	30.32 ± 9.05	40.92 ± 7.44	43.11 ± 5.12	43.89 ± 5.94	41.13 ± 4.97	31.43 ± 2.60	31.38 ± 1.80	18.07 ± 2.80	34.84 ± 9.08
	Bioaccessibility	46.46 ± 4.51	35.56 ± 5.32	33.52 ± 5.06	48.31 ± 3.23	43.28 ± 4.65	26.45 ± 3.75	38.93 ± 7.74	45.24 ± 4.43	46.37 ± 5.86	38.20 ± 3.13	29.75 ± 3.01	29.40 ± 5.11	16.88 ± 1.87	34.31 ± 10.24

	Micellar efficiency	101.03 ± 13.69	101.32 ± 9.41	88.54 ± 14.12	93.19 ± 10.80	103.59 ± 16.97	78.75 ± 14.42	94.40 ± 8.73	98.23 ± 8.38	97.12 ± 13.42	89.21 ± 10.84	95.05 ± 9.80	90.72 ± 13.72	92.30 ± 10.44	93.77 ± 3.29
γT3	Stability	122.93 ± 15.11	83.73 ± 9.52	ND	ND	ND	ND	34.44 ± 50.01	ND	109.27 ± 34.51	ND	99.33 ± 8.04	ND	94.49 ± 30.17	50.52 ± 50.70
	Solubility	65.23 ± 9.65	34.22 ± 5.14	ND	ND	ND	ND	16.57 ± 25.09	ND	63.05 ± 5.39	ND	56.80 ± 5.47	ND	54.24 ± 10.23	29.02 ± 29.31
	Bioaccessibility	59.08 ± 12.94	38.86 ± 5.66	ND	ND	ND	ND	16.32 ± 23.81	ND	58.95 ± 13.21	ND	56.34 ± 5.00	ND	47.14 ± 6.79	27.07 ± 27.31
	Micellar efficiency	93.56 ± 26.13	102.38 ± 9.89	ND	ND	ND	ND	32.66 ± 46.25	ND	82.31 ± 7.84	ND	99.49 ± 7.11	ND	85.38 ± 14.15	44.53 ± 44.84
δT3	Stability	122.49 ± 29.17	86.47 ± 14.92	114.30 ± 16.97	140.81 ± 7.79	102.99 ± 23.60	92.59 ± 22.06	109.94* ± 18.39	87.37 ± 13.62	74.11 ± 5.05	66.82 ± 8.68	58.19 ± 5.82	93.92 ± 11.37	79.80 ± 13.59	76.70* ± 12.02
	Solubility	42.94 ± 8.26	35.29 ± 10.89	9.69 ± 2.11	76.13 ± 13.61	67.80 ± 11.69	19.74 ± 6.32	41.93 ± 23.86	71.18 ± 11.24	30.71 ± 9.84	27.80 ± 6.44	18.86 ± 4.46	44.96 ± 5.76	25.78 ± 4.35	36.55 ± 17.37
	Bioaccessibility	39.28 ± 4.64	38.89 ± 7.14	7.46 ± 0.90	51.80 ± 3.63	61.76 ± 6.58	25.98 ± 3.50	37.53 ± 17.48	58.06 ± 3.58	20.78 ± 3.46	27.79 ± 6.79	16.99 ± 2.37	36.16 ± 6.04	30.71 ± 9.84	30.86 ± 13.55
	Micellar efficiency	93.58 ± 13.95	89.65 ± 18.77	79.76 ± 6.52	67.21 ± 10.79	94.06 ± 19.41	101.92 ± 23.19	86.20 ± 12.34	85.17 ± 14.89	76.91 ± 18.14	90.29 ± 16.64	83.21 ± 14.20	73.53 ± 15.98	92.76 ± 10.73	83.65 ± 6.80

αT: α-tocopherol; βT: β-tocopherol; γT: γ-tocopherol; δT: δ-tocopherol; αT3: α-tocotrienol; βT3: β-tocotrienol; γT3: γ-tocotrienol; δT3: δ-tocotrienol; ND: not detected. *Mean values differ significantly ($p<0.05$) between bitter and non-bitter seeds.

Table S6. Mean (± standard deviation) polydispersity index, particle size (ζ average) and surface charge (ζ potential) of mixed micelles of digested quinoa

	Bitter						Non-bitter					
	CHEN-160	CHEN-389	PI-614885	PI-614888	PI-634924	PI-665276	Ames-13751	CHEN-126	CHEN-159	CHEN-465	D-12184	PI-510549
PDI	0.63 ± 0.01	0.58 ± 0.03	0.51 ± 0.02	0.63 ± 0.03	0.66 ± 0.02	0.41 ± 0.09	0.50 ± 0.05	0.63 ± 0.09	0.46 ± 0.01	0.34 ± 0.19	0.58 ± 0.07	0.59 ± 0.14
ζ average [nm]	169.71 ± 7.67	119.42 ± 3.32	129.18 ± 5.12	146.29 ± 2.19	153.69 ± 11.72	155.38 ± 13.47	190.26 ± 48.49	269.12 ± 32.06	142.95 ± 10.85	289.58 ± 13.02	115.96 ± 15.00	217.31 ± 94.96
ζ potential [mV]	-25.96 ± 1.07	-26.47 ± 0.33	-26.46 ± 0.31	-26.14 ± 0.45	-26.47 ± 0.56	-26.60 ± 0.78	-26.64 ± 1.19	-28.79 ± 0.48	-26.27 ± 0.33	-30.39 ± 0.86	-25.34 ± 1.93	-24.00 ± 7.19
